# O04 Use of the novel antifungal rezafungin in outpatient parenteral antibiotic therapy: early experience from a single centre

**DOI:** 10.1093/jacamr/dlae217.004

**Published:** 2025-01-23

**Authors:** H Davidson, I Dunstan, T Yau, A Houston, M Basarab, T Bicanic

**Affiliations:** Institute of Infection and Immunity, City St George’s University of London, London, UK; St George’s University Hospital, London, UK; St George’s University Hospital, London, UK; St George’s University Hospital, London, UK; St George’s University Hospital, London, UK; St George’s University Hospital, London, UK; Institute of Infection and Immunity, City St George’s University of London, London, UK; St George’s University Hospital, London, UK

## Abstract

**Background:**

Rezafungin is a novel long-acting echinocandin antifungal drug with weekly dosing, which was licensed for treatment of invasive candidiasis in the UK in January 2024. We report on the early use of rezafungin in our outpatient parental antibiotic therapy (OPAT) service.

**Objectives:**

To review indications, treatment regimens, outcomes and adverse events in adult patients receiving rezafungin at a tertiary infectious disease centre.

**Methods:**

All adult patients who received rezafungin therapy via the OPAT service since it was added to the hospital formulary in July 2024 were included. Patient demographics, infection diagnosis, treatment regimens and outcomes were recorded. A single dose of rezafungin was assumed to be equivalent to seven doses of a comparable echinocandin (e.g. anidulafungin).

**Results:**

Five patients (age range 30–80 years) received rezafungin therapy between July 2024 and October 2024. A total of 18 doses were administered. The median course length was 4 doses (range: 2–5). One patient received two courses of rezafungin as part of a planned series, otherwise patients received a single course. Indications for treatment were *Candida* osteomyelitis, recurrent mucocutaneous candidiasis (3 oral/oesophageal, 2 resistant or refractory to fluconazole; 1 drug interactions), and pulmonary aspergilloma (cyclical use: 2 weeks’ course every 8 weeks, in combination with voriconazole due to progression on monotherapy). Reasons for choosing rezafungin over daily echinocandins were patient preference/convenience, facilitating earlier patient discharge (1 case) and cost effectiveness compared with the cost of daily caspofungin administration by the OPAT nurses. Underlying conditions predisposing to fungal infection were diabetes mellitus (3), immunosuppression secondary to liver transplantation, STAT1 gain-of-function mutation and multisystem sarcoidosis on long-term prednisolone. All patients with mucocutaneous candidiasis reported a treatment response; treatment in the patient with aspergilloma and the patient with osteomyelitis is ongoing. Regarding treatment-emergent adverse reactions: there were no cases of pyrexia, hypokalaemia, pneumonia or septic shock (the most common AEs in reported in the RESTORE trial). One hundred and nine doses of equivalent echinocandin were saved through once weekly dosing.

**Conclusions:**

Early use of rezafungin at our centre suggests it is a well-tolerated, convenient and useful addition to the antifungal armamentarium, particularly in the outpatient setting. Monitoring of blood tests is essential, particularly haemoglobin, renal function and electrolytes, including magnesium.

